# An experimental challenge model for infecting African catfish (*Clarias gariepinus*) with *Edwardsiella tarda*

**DOI:** 10.17221/102/2025-VETMED

**Published:** 2026-07-24

**Authors:** Ivona Toulova, Vera Vaibarova, Ivana Papezikova, Katerina Matejickova, Ivana Mikulikova, Hana Novotna, Miroslava Palikova

**Affiliations:** ^1^Department of Ecology and Diseases of Zoo Animals, Game, Fish and Bees, Faculty of Veterinary Hygiene and Ecology, University of Veterinary Sciences Brno, Brno, Czech Republic; ^2^Department of Zoology, Fisheries and Hydrobiology, Faculty of AgriSciences, Mendel University in Brno, Brno, Czech Republic; ^3^Department of Infectious Diseases and Microbiology, Faculty of Veterinary Medicine, University of Veterinary Sciences Brno, Brno, Czech Republic; ^4^Department of Infectious Diseases and Preventive Medicine, Veterinary Research Institute, Brno, Czech Republic

**Keywords:** bacterial infection, edwardsiellosis, immersion bath, intraperitoneal injection

## Abstract

Abstract: *Edwardsiella tarda* is an important fish pathogen that causes outbreaks leading to significant losses in aquacultural species such as African catfish (*Clarias gariepinus*). In order to study host-pathogen interactions and design preventive strategies, it is essential to prepare a reliable experimental infection model. The aim of this study was to develop a reproducible immersion challenge model for *E. tarda* infection in *C. gariepinus*. The present study consisted of preliminary experiments and a main experiment evaluating the different routes of infection and the bacterial concentrations and exposure durations in an immersion bath. Preliminary experiments demonstrated that infection could be induced by either intraperitoneal injection or immersion bath, and that infection was more successful at higher concentrations. The main experiment involved two concentrations (10^8^ and 2 × 10^8^ CFU/ml) of immersion bath and two immersion durations (1 and 2 h), each repeated twice. The fish were monitored for clinical signs and mortality throughout the experiment and samples were collected on day 10 post-infection for haematological, immunological, and pathological assessment. High-dose immersion reliably induced infection, with disease progression depending on bacterial concentration and exposure duration. This established model is a practical and reproducible tool for future studies on the pathogenesis and immunity of* C. gariepinus*, as well as the use of immunostimulants and therapeutics*.*

African catfish (*Clarias gariepinus*) is a species that is widely distributed across Africa and has now been introduced to Asia, Europe (Worldfish Center FishBase Project Team 2003) and Brazil, where it is considered an invasive species (Vitule et al. 2006). Due to its fast growth, high stocking density and tolerance to environmental extremes, it has become an important fisheries and aquaculture species (Hossain et al. 1998; Van de Nieuwegiessen et al. 2009). However, the rapid expansion of aquaculture is also increasing the risk of disease outbreaks. Intensive farming practices, such as high stocking densities, frequent translocation of fish and reduced genetic diversity, provide ideal conditions for pathogens to proliferate (Bondad-Reantaso et al. 2005).

*Edwardsiella tarda*, a motile, rod-shaped, Gram-negative, facultatively anaerobic bacterium of the family Enterobacteriaceae, is widely distributed in aquatic environments such as lakes, streams and seawater, as well as mud, and can infect both freshwater and marine fish, as well as humans. It is an intracellular pathogen that causes a disease called edwardsiellosis (Mohanty and Sahoo 2007; Park et al. 2012; Xu and Zhang 2014). Since the first reported outbreak of eel mortality in 1989 (Han et al. 1989), this bacterium has been responsible for significant economic losses in aquaculture.

Edwardsiellosis is a systemic infection characterised by extensive lesions in the skin, muscles and internal organs, as well as exophthalmia and ascites. It affects various species of fish, including eels (Anguilliformes), catfish (Siluriformes), mullet (Mugilidae), chinook salmon (*Oncorhynchus tshawytscha***)**, flounder (Pleuronectoidei), carp (*Cyprinus carpio*) and tilapia (Cichlidae) (Mohanty and Sahoo 2007; Park et al. 2012). Mortality rates can be high among infected fish, particularly turbot, for which mortality rates of up to 95% in adults and 80% in juveniles have been reported (Xu and Zhang 2014). Edwardsiellosis in *C. gariepinus* leads to loss of pigmentation, swelling of the abdomen and petechial haemorrhages in the fins, bloody ascites in the abdomen and inflamed liver, spleen and kidneys (Abraham et al. 2015). Although *E. tarda* can become non-culturable in adverse conditions, it remains viable and is capable of resuming pathogenic activity once conditions improve, emphasising its persistence as a threat to aquaculture (Du et al. 2007).

Here, we present the development and optimisation of a reproducible infection model for *E. tarda* in African catfish. This will enable future research into the disease, immunity, and the use of immunostimulants and therapeutics in this species.

## MATERIAL AND METHODS

### Ethical statement

The experiment was performed in compliance with the Czech law for the Protection of Animals against Cruelty, as approved by the Ethical Committee of the University of Veterinary Sciences Brno, Czech Republic (MSMT-1688/2024-7).

### Experimental fish

African catfish weighing 94.85 ± 74.62 g were obtained from the fish breeding facility at Mendel University in Brno, Czech Republic, where bacteriological and parasitological examination is performed in connection with fish transfers and *E. tarda* has not been detected for a prolonged period. The fish were placed in 60 l glass tanks connected as a recirculating aquaculture system and left to acclimatise for three days. Throughout the acclimation period and the experimental procedure, the fish were fed a commercial catfish diet (Skretting Protec MP, Fontaine-les-Vervins, France) at a rate of 2% of their body weight per day. Feeding ceased one day before the challenge, and a small number of pellets were provided a few hours after the challenge to evaluate feeding behaviour.

### Bacterial strains

The *E. tarda* strain used in the present study was isolated from the head kidneys of naturally infected farmed African catfish. This *E. tarda* 968 1L isolate was revived from glycerol cryostock and cultivated on Columbia blood agar containing 3.9% Columbia blood agar base (Oxoid, Basingstoke, UK) and 5% defibrinated sheep blood at 22 °C for 48 h, after which the isolate was sub-cultivated at 22 °C for 24 h, prior to analysis. A single isolated colony was then resuspended in brain heart infusion (BHI) broth (Oxoid, Basingstoke, UK) and incubated at 22 °C and at 160 rpm for 18 hours. After incubation, the culture was centrifugated at 2 500 *g* for 10 minutes.

For intraperitoneal application, the centrifuged culture was resuspended in phosphate-buffered saline (PBS) to a final turbidity of 1.0 °McFarland, equivalent to approximately 10^8^ bacterial cells/ml. The bacterial suspension was subsequently diluted in sterile PBS to achieve final concentrations of 10^4^ and 10^5^ CFU/ml.

For the immersion bath, the centrifuged culture was resuspended in PBS until it reached a final turbidity of 6.0 °McFarland, equivalent to approximately 10^9^ bacterial cells/ml. The final bacterial concentrations used for the immersion bath (10^6^, 10^7^ and 10^8^ cells/ml) were obtained by diluting the 10^9^ cells/ml stock suspension with an appropriate amount of water. The accuracy of bacterial concentration and suspension purity were confirmed through standard plate culture.

### Experimental design

A consistent challenge model of *E. tarda* infection in African catfish was developed and refined through two preliminary experiments and one main experiment. Two infection methods were evaluated in the preliminary experiments to determine whether they could induce the disease. Different bacterial concentrations for immersion were subsequently tested to identify a dose capable of producing consistent clinical signs while maintaining manageable mortality, and to confirm the reproducibility of the infection outcome. The main experiment then evaluated the effects of different bacterial concentrations and immersion durations on the development of infection in the African catfish.

### Preliminary experiments

The preliminary experiments (P1 and P2) investigated whether *E. tarda* infection could be reliably introduced to African catfish via an immersion bath (IB) or intraperitoneal (i.p.) injection.

For experiment P1, the fish were divided into five groups of 10 individuals each. Three of these groups were subsequently challenged via IB for one hour using bacterial suspensions at concentrations of 10^6^, 10^7^ and 10^8^ CFU/ml, respectively. A further two groups were exposed via i.p. injection with 0.1 ml of bacterial suspension at concentrations of 10^3^ and 10^4^ CFU per fish, respectively. During the experiment, the water temperature was maintained at 25.8 ± 0.5 °C and the pH at 7.8 ± 0.8. The fish were monitored twice a day for clinical signs, behavioural changes and mortalities. As fish were maintained in groups without individual identification, assessment of clinical signs was limited to general observations at the group level. Experiment P1 ended 10 days post-infection (DPI). Nitrogenous compounds were measured twice weekly throughout the P1, during which monitoring revealed elevated nitrite levels associated with suboptimal water quality. Based on observations from this part, the IB experiment was repeated under improved water quality conditions, with nitrogenous compounds measured more frequently.

In experiment P2, fish were divided into three groups of 10 individuals each and exposed to *E. tarda* via IB for one hour at concentrations of 10^6^, 10^7^ and 10^8^ CFU/ml, respectively. Following from P1, the biofilter capacity was increased to prevent high levels of nitrogenous compounds. During the experiment, the water temperature was maintained at 25.4 ± 0.5 °C, the pH at 7.3 ± 0.8, and the fish were monitored twice a day for clinical signs. Nitrogenous compounds were measured every day. Due to the absence of clinical signs at 6 DPI, this stage of the experiment was terminated. The fish were euthanised with a blow to the head, followed by rapid spinal transection, and bacterial cultivation was performed on the head kidneys of 3 fish from the 10^8^ group to confirm or exclude infection. The remaining groups were not tested, as the absence of clinical signs suggested an unsuccessful challenge, and based on experience from P1, the 10^8^ concentration was considered the most likely to allow detection of *E. tarda*.

Similarly, head kidneys of ten deceased fish (mortalities) from P1 experiment were sampled for bacteriological analysis to confirm the presence of *E. tarda.* However, bacteriological cultivation could not be performed on 18 dead fish, as post-mortem cannibalism caused extensive damage to the body surface and internal organs, making the samples unsuitable for sterile sampling. At the end of P1, the surviving fish were not subjected to bacteriological examination, as the experiment was terminated due to elevated nitrite levels.

Bacteriological cultivation was performed on Columbia blood agar prepared with 3.9% Columbia blood agar base (Oxoid, Basingstoke, UK) supplemented with 5% defibrinated sheep blood, and incubated at 22 °C for 24 hours. The bacterial colonies grown were identified using matrix-assisted laser desorption/ionisation mass spectrometry (MALDI TOF MS; Bruker Daltonics, Bremen, Germany), in accordance with the manufacturer’s instructions (Bruker Daltonik GmbH 2011).

### Main experiment

The main experiment examined the effect of different bacterial concentrations and IB durations on the progression of *E. tarda* infection in African catfish. Five experimental groups, each consisting of 15 fish and set up in duplicate, were treated as follows: 10^8^ CFU/ml for one hour (10^8^ 1 h), 10^8^ CFU/ml for two hours (10^8^ 2 h), 2 × 10^8^ CFU/ml for one hour (2 × 10^8^ 1 h) and 2 × 10^8^ CFU/ml for two hours (2 × 10^8^ 2 h), with a fifth group serving as a control.

The control group was included in the recirculating system, and therefore was unintentionally exposed to background bacterial levels, and was also exposed to a bath containing broth without bacteria for two hours. During the experiment, the water temperature was maintained at 25.9 ± 0.4 °C, the pH at 7.7 ± 0.4, and the fish monitored twice a day for clinical signs, behavioural changes and mortalities. At 10 DPI, four fish were randomly selected from each tank (eight fish per group) and blood was collected via caudal vein puncture using sterile heparinised syringes for the determination of red and white blood cell counts, haematocrit values and specific antibody titres. Fish were sampled without sedation and gently restrained in a moist cloth. A volume of 1.5–2 ml of blood was collected per fish.

The fish were then euthanised by a blow to the head followed by rapid spinal dislocation. Blood cell count and haematocrit values were determined according to Svobodova et al. (2012). They were then measured and weighed, after which a necropsy was performed to assess gross pathological changes. Finally, bacterial cultivation was performed on the head kidneys to confirm the presence of infection. Bacteria were cultivated and identified in the same way as in experiments P1 and P2.

Specific anti-*E. tarda* antibodies in catfish serum were detected using an indirect ELISA with a monoclonal antibody against carp immunoglobulin (MAb anti-CIg 1E10/2A8; Vesely et al. 2006). ELISA plates were coated with sonicated *E. tarda* antigen (1 μg/ml) in carbonate–bicarbonate buffer and incubated overnight at 4 °C. After blocking with 5% FBS in PBST, serial dilutions (1 : 200–1 : 1 600) of pre- and post-immunisation sera were applied and incubated at 37 °C for 1 hour. Bound carp Ig was detected with MAb anti-CIg followed by rabbit anti-mouse IgG conjugated to horseradish peroxidase. The reaction was visualised with tetramethylbenzidine substrate, and absorbance was measured at 450 nm.

### Statistical analysis

All statistical analyses were performed using the Statistica v14 statistical package (TIBCO Software Inc. 2020, USA). Significant differences in the data were assessed using one-way ANOVA followed by Tukey post-hoc tests. In all cases, the significance level was set at *P *< 0.05.

## RESULTS

### Preliminary experiments

**Figure 1:**
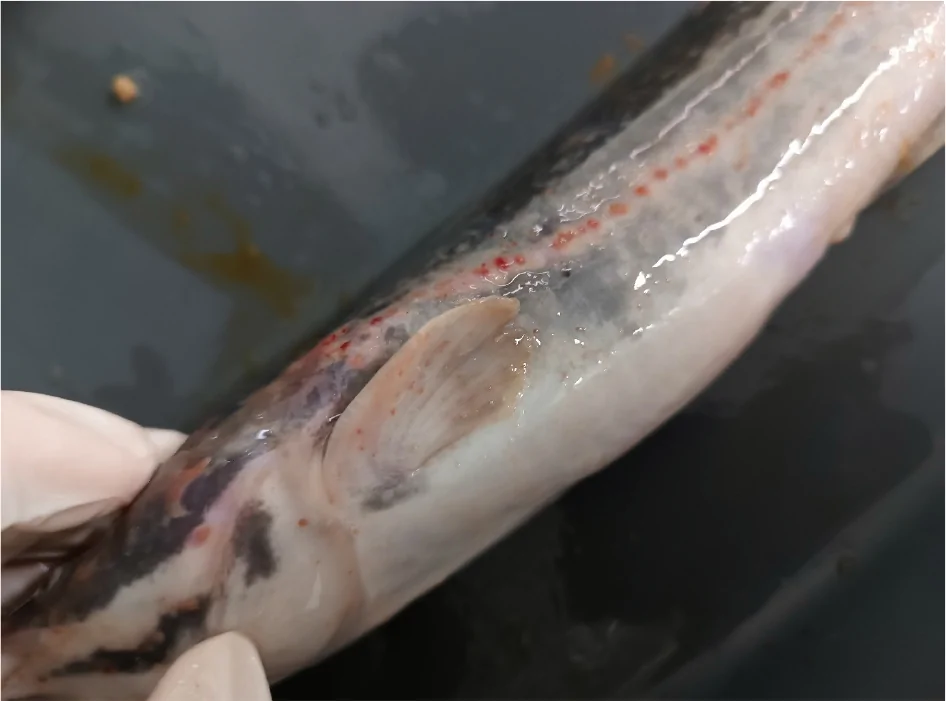
Changes in the lateral line of African catfish experimentally infected with *Edwardsiella tarda*, showing haemorrhagic and enlarged lateral line pores

**Figure 2:**
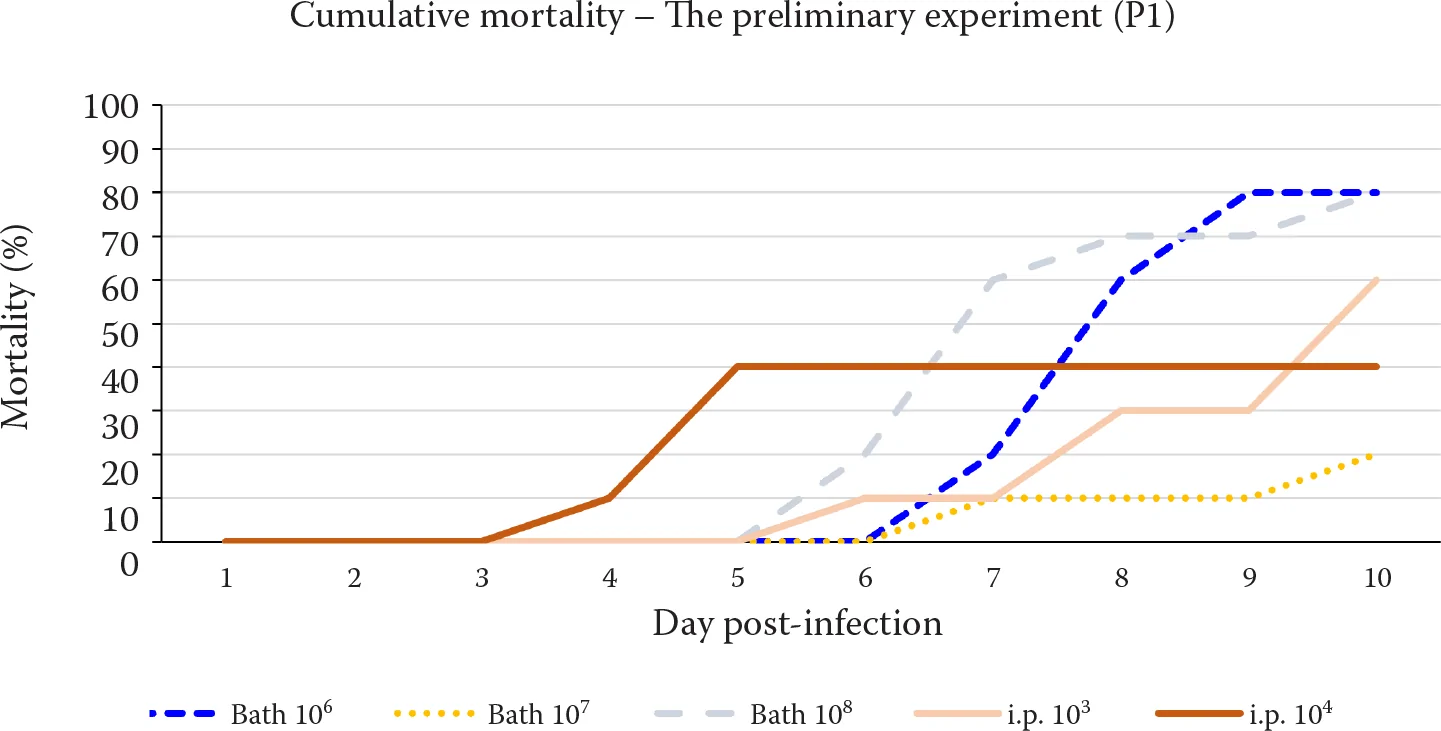
Cumulative mortality of African catfish challenged with *Edwardsiella tarda* via immersion bath (Bath) or intraperitoneal injection (i.p.) at different bacterial concentrations

P1 – The first clinical sign observed in all fish was loss of appetite (inappetence) at 2 and 3 DPI, after which, the fish resumed normal feeding. Changes to the lateral line, including haemorrhaging and enlarged pores, were observed at 6–8 DPI on several fish from the IB 10^8^ and i.p. 10^4^ groups (Figure 1), these changes subsequently resolved and were absent in all fish at 10 DPI. Mortality began at 4 DPI and increased rapidly between 6 and 10 DPI. The highest mortality occurred in the Bath 10^6^ and Bath 10^8^ groups, and the lowest in the Bath 10^7^ group (Figure 2). Increased concentrations of nitrogenous compounds (NO_2_^–^ > 30 mg/l) were observed. Bacteriological cultivation of head kidney was performed in ten deceased fish across all the experimental groups and the presence of *E. tarda* was confirmed in four fish from the Bath 10^8^ group and one fish from i.p. 10^3^ group, the remaining five tested fish were *E. tarda* negative.

P2 – None of the fish showed any clinical signs, such as inappetence, behavioural changes or skin lesions. Likewise, no mortalities were recorded throughout the experiment. Bacteriological cultivation confirmed the presence of *E. tarda* in all three euthanised fish selected for bacteriological examination from the Bath 10^8^ group.

### Main experiment

The first clinical signs manifested as abnormal swimming behaviour, characterised by a head-up, tail-down movement. This was initially observed in groups 10^8^ (2 h), 2 × 10^8^ (1 h), and 2 × 10^8^ (2 h) at 2 DPI, and subsequently occurred sporadically across all other groups on different days. Lateral line alterations, including haemorrhaging and enlarged pores, were observed in several fish at 4 DPI in groups 10^8^ (1 h) and 2 × 10^8^ (1 h), and enlarged head kidneys were observed in one fish at 5 DPI in group 10^8^ (1 h).

**Figure 3:**
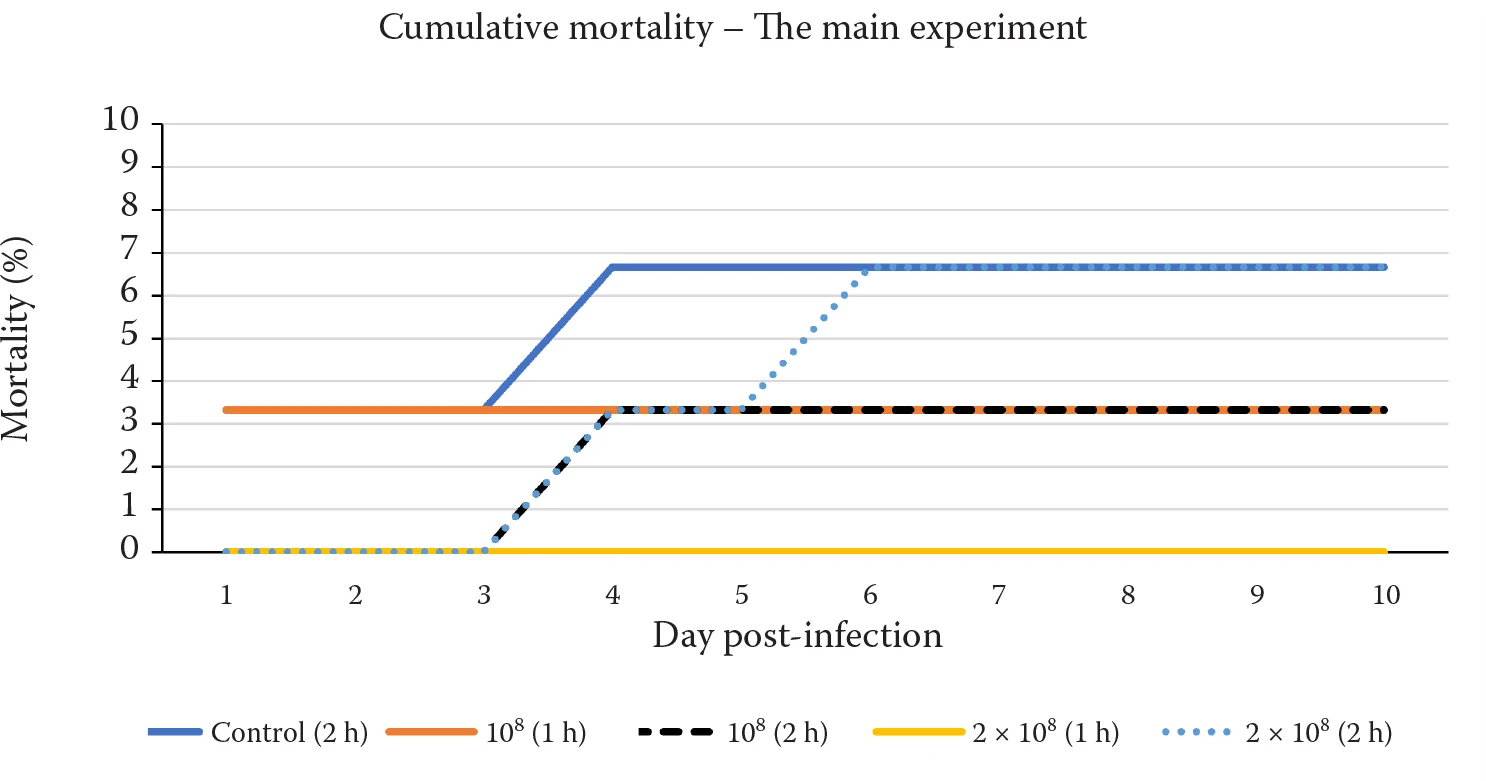
Cumulative mortality of African catfish challenged with *Edwardsiella tarda* via immersion bath for different durations and at different bacterial concentrations

Cumulative mortality remained low in all groups (Figure 3). The first mortalities in the control group and in group 10^8^ (1 h) occurred on the day of infection. The highest mortality rate was recorded in the control group (two fish), whereas no deaths were observed in the 2 × 10^8^ (1 h) group. Bacteriological examination confirmed the presence of *E. tarda* in two deceased fish – one from group 10^8^ (2 h) and one from group 2 × 10^8^ (2 h). Other dead fish were not suitable for bacteriological cultivation due to severe tissue damage caused by cannibalism. During the sampling performed at the end of the experiment, *E. tarda* was detected from only two of the 40 examined fish, originating from groups 10^8^ (2 h) and 2 × 10^8^ (2 h).

Haematocrit values were significantly lower in the group exposed at 2 × 10^8^ (2 h) than in the control group and 10^8^ (1 h) group (Table 1). The specific antibody titre was significantly higher in all infected groups compared to the control. Additionally, significant differences were observed between groups 10^8^ (2 h) and 2 × 10^8^ (2 h), and between 2 × 10^8^ (1 h) and 2 × 10^8^ (2 h). No significant differences were observed between groups in other haematological parameters, such as red and white blood cell counts and differential leukocyte counts.

**Table 1:** Haematological and immunological parameters of fish in different experimental groups at 10 days post-infection with *Edwardsiella tarda*

Parameter (unit)	Control (2 h)	10^8^ CFU/ml (1 h)	10^8^ CFU/ml (2 h)	2 × 10^8^ CFU/ml (1 h)	2 × 10^8^ CFU/ml (2 h)
RBC (10^12^/l)	1.49 ± 0.15	1.38 ± 0.20	1.45 ± 0.18	1.40 ± 0.24	1.34 ± 0.15
WBC (10^9^/l)	74.63 ± 22.85	73.38 ± 12.13	63.50 ± 17.05	66.88 ± 14.39	54.88 ± 15.32
Hct (%)	24.44 ± 2.32^a^	24.44 ± 1.08^a^	23.44 ± 2.16^ab^	23.06 ± 2.93^ab^	21.38 ± 1.62^b^
Lymphocytes (%)	91.75 ± 2.55	89.25 ± 3.99	90.25 ± 5.99	92.88 ± 3.04	87.25 ± 3.85
Neutrophils (%)	5.88 ± 3.18	7.38 ± 3.81	7.75 ± 5.85	5.38 ± 2.56	10.50 ± 3.78
Monocytes (%)	2.38 ± 1.06	3.38 ± 2.62	2.00 ± 0.93	2.00 ± 1.07	2.25 ± 0.89
PA integral (RLU.min)	20 415 ± 13 946	17 119 ± 28 688	4 407 ± 3 809	5 709 ± 3 304	23 251 ± 21 485
PA peak-time (min)	96.75 ± 7.81	97.88 ± 11.56	99.00 ± 9.07	92.25 ± 12.19	87.75 ± 14.14
PA peak (RLU)	331.75 ± 181.18	263.19 ± 406.82	82.13 ± 73.21	104.31 ± 56.71	393.00 ± 358.43
Specific antibodies (abs. at 450 nm)	0.53 ± 0.29^a^	1.29 ± 0.36^bc^	1.24 ± 0.28^b^	1.12 ± 0.32^b^	1.69 ± 0.29^c^

^a–c^Different superscripts within a row denote significance (*P* < 0.05); Values are presented as mean ± SD

abs. = absorbance; CFU = colony-forming unit; Hct = haematocrit; PA = phagocytic activity; SD = standard deviation; RBC = red blood cell count; RLU = relative light unit; WBC = white blood cell count

Only mild gross pathological changes were observed in most of the fish. These included a pale or fragile liver, and a small amount of fluid present in the body cavity. During the experiment, the fish gradually recovered, as indicated by the gradual disappearance of external signs such as lateral line changes.

## DISCUSSION AND CONCLUSION

Immersion bath exposure was investigated as an experimental infection route, as it closely mimics the natural entry of *E. tarda*. Intraperitoneal injection was included in the initial experimental stage (P1) primarily as a reference method in case bath exposure was unsuccessful. Signs of infection following intraperitoneal injection, including transient inappetence, lateral line alterations, and recovery of *E. tarda* from the head kidney of one examined fish, suggest that this route was effective. However, once infection could be induced via IB, further experiments focused on this approach due to its greater relevance to natural infection conditions.

In preliminary experiment P1, both infection routes led to fish mortality, with highest mortality observed in IB groups exposed to 10^6^ and 10^8^ CFU/ml. However, mortality in these groups may have been influenced by increased nitrogenous compound concentrations in the water (NO_2_^–^ > 30 mg/l). This assumption is supported by the results of the preliminary experiment P2, where, after increasing the biofilter capacity and stabilising water quality, no clinical signs or mortality were observed under the highest bacterial concentration (10^8^ CFU/ml), despite presence of *E. tarda* being confirmed through bacteriological cultivation of head kidney in this group. This suggests that the bacteria were able to infect the fish without causing acute disease, implying that the IB infection route can lead to subclinical infection.

The main experiment demonstrated that *E. tarda* infection in African catfish via IB is reproducible and induces mild clinical signs. The disease was characterised by consistent, low-level mortality, which was already observed at DPI 1. This early onset of mortality is somewhat unexpected and raises the question of whether the disease progression itself was truly so rapid. However, mortality observed in the non-challenged control group indicates that the early losses may have resulted from a combination of stress-related factors, including handling during immersion, the immersion procedure itself, and cannibalism which is common in this fish species. Clinical signs included abnormal swimming behaviour (head-up tail-down movement) and changes to the lateral line, manifested as enlarged, haemorrhagic lateral line pores. These signs were primarily observed in groups that were exposed to higher concentrations of bacteria for longer periods of time, though they gradually disappeared over the course of a few days, suggesting that the fish were able to overcome the infection. This interpretation is further supported by the significantly increased specific antibody titres observed in infected groups, indicating effective activation of the adaptive immune response and subsequent control of the infection. The largely unchanged haematological profile further indicates that the infection proceeded without severe systemic impairment. However, the reduced haematocrit observed in the group 2 × 10^8^ (2 h) may reflect a transient stress response associated with the higher bacterial load, consistent with findings in *C. gariepinus* where acute stress from transportation and handling was associated with lower haematocrit values compared to non-stressed fish (Olopade et al. 2022).

The severity of the clinical signs observed in this study differed from those reported after injection application in previous studies, where authors noted skin lesions, petechial haemorrhages, cutaneous ulcers and fin and tail rot, along with high mortality rates (Abo El-Yazeed and Ibrahem 2009; Abraham et al. 2015). Abraham et al. (2015) also reported 100% mortality at 7 DPI following intramuscular application. These differences could be explained by the distinct pathways of pathogen entry, whereby infection via injection bypasses the natural epithelial barriers and induces an acute systemic infection, whereas IB exposure simulates natural entry via the skin and gills. This leads to a milder course of infection (at the concentrations tested) that likely reflects the natural route of infection under aquacultural conditions.

An interesting finding was the change in the lateral line, which may be associated with local epithelial injury or an inflammatory response by immune cells in the skin. This should be investigated further in future histological studies. Corrales et al. (2009) reported similar changes to the lateral line in channel catfish (*Ictalurus punctatus*), though without haemorrhaging. The authors characterised the changes as lateral line depigmentation and epidermal thinning in the area surrounding the neuromasts. They also noted that the changes were associated with nutritional stress induced by several months of starvation. When environmental conditions improved, the lesions were seen to resolve spontaneously. A similar progression was observed in the present study, where the enlargement and haemorrhaging of the lateral line pores disappeared within a few days.

Our results also suggest that water quality could significantly impact the progression of the disease. In the P1 experiment, the increased mortality observed was likely influenced by elevated concentrations of nitrogenous compounds. This finding is consistent with the observations of Ajani and Adeyemo (2012), who reported that nitrite exposure may induce physiological stress and impair fish condition, particularly under suboptimal or combined stress conditions. Stabilisation of the water environment in subsequent experiments (P2) resulted in a marked reduction in mortality and a milder course of the disease.

It should be noted that the observation of clinical signs was based on visual monitoring of the fish. While this approach enabled us to track the disease’s general progression, it did not allow us to assess how pathological changes developed, progressed or resolved over time in detail. Several clinical signs, particularly changes to the lateral line, appeared and disappeared rapidly. Without intermediate sampling, such as histology and bacteriology at defined time points, it is impossible to determine their cause or how they change over time. Additional sampling time points would be beneficial for future experiments, as they would allow the development of both external and internal pathological changes to be captured, and their relationship to the course of *E. tarda* infection to be clarified.

This study demonstrated that African catfish are highly tolerant of *E. tarda* infection*.* High concentrations of the pathogen are required to induce disease, and even in these cases, clinical signs disappeared within a few days, and mortality was very low. Nevertheless, infection can be successfully established via IB, as confirmed by bacterial cultivation from the head kidney and an increase in specific antibodies. The IB route was thus proven to be a suitable, gentle and reproducible method of experimentally infecting African catfish with *E. tarda.* The model reliably induces measurable physiological and immunological responses without causing significant mortality. This makes it suitable for further studies focusing on pathogenesis, strain virulence assessment, immunostimulant testing or vaccine development.

## References

[R1] Abo El-Yazeed H, Ibrahem MD. Studies on Edwardsiella tarda infection in catfish and Tilapia nilotica. J Vet Med Res. 2009;19(1):44-50.

[R2] Abraham TJ, Mallick PK, Adikesavalu H, Banerjee S. Pathology of Edwardsiella tarda infection in African catfish, Clarias gariepinus (Burchell 1822), fingerlings. Arch Pol Fish. 2015;23(3):141-8.

[R3] Ajani F, Adeyemo OK. Nitrite intoxication of Clarias gariepinus at different water temperatures. Int J Fish Aquacult. 2012 Mar;4(4):77-80.

[R4] Bondad-Reantaso MG, Subasinghe RP, Arthur JR, Ogawa K, Chinabut S, Adlard R, Tan Z, Shariff M. Disease and health management in Asian aquaculture. Vet Parasitol. 2005 Sep 30;132(3-4):249-72. 16099592 10.1016/j.vetpar.2005.07.005

[R5] Bruker Daltonik GmbH. MALDI Biotyper 3.0 user manual. Rev. 2. Bremen: Bruker Daltonik; 2011.

[R6] Corrales J, Ullal A, Noga EJ. Lateral line depigmentation (LLD) in channel catfish, Ictalurus punctatus (Rafinesque). J Fish Dis. 2009 Aug;32(8):705-12. 19531093 10.1111/j.1365-2761.2009.01069.x

[R7] Du M, Chen J, Zhang X, Li A, Li Y, Wang Y. Retention of virulence in a viable but nonculturable Edwardsiella tarda isolate. Appl Environ Microbiol. 2007 Feb;73(4):1349-54. 17189433 10.1128/AEM.02243-06 PMC1828651

[R8] Han X, Li W, Cheng G. Study on the edwardsielliasis of the eel. Acta Hydrobiol Sin. 1989;13(3):259-64.

[R9] Hossain MAR, Beveridge MCM, Haylor GS. The effects of density, light and shelter on the growth and survival of African catfish (Clarias gariepinus Burchell, 1822) fingerlings. Aquaculture. 1998 Jan 30;160(3-4):251-8.

[R10] Mohanty BR, Sahoo PK. Edwardsiellosis in fish: A brief review. J Biosci. 2007 Dec;32(7):1331-44. 18202458 10.1007/s12038-007-0143-8

[R11] Olopade OAA, Dienye HE, Agbo KK. Haematological profile of catfish (Clarias gariepinus Burchell, 1822) following the stress associated with transportation and handling in the market. J Faculty Food Eng. 2022 Apr;21(3):230-6.

[R12] Park SB, Aoki T, Jung TS. Pathogenesis of and strategies for preventing Edwardsiella tarda infection in fish. Vet Res. 2012 Oct 4;43(1):67. 23035843 10.1186/1297-9716-43-67 PMC3479428

[R13] Svobodova Z, Pravda D, Modra H. Metody hematologickeho vysetrovani ryb [Methods of haematological examination of fish] [Internet]. Edice metodik, vol. 122. Vodnany: Research Institute of Fish Culture and Hydrobiology; 2012. 38 p. Czech.

[R14] Van de Nieuwegiessen PG, Olwo J, Khong S, Verreth JAJ, Schrama JW. Effects of age and stocking density on the welfare of African catfish, Clarias gariepinus Burchell. Aquaculture. 2009 Mar;288(1-2):69-75.

[R15] Vesely T, Reschova S, Pokorova D, Hulova J, Nevorankova Z. Production of monoclonal antibodies against immunoglobulin heavy chain in common carp (Cyprinus carpio L.). Vet Med-Czech. 2006 Mar;51(5):296-302.

[R16] Vitule JRS, Umbria SC, Aranha JMR. Introduction of the African catfish Clarias gariepinus (Burchell, 1822) into Southern Brazil. Biol Invasions. 2006 Jun;8(4):677-81.

[R17] Worldfish Center FishBase Project Team. FishBase species profile: Clarias gariepinus (Burchell, 1822) North African catfish. Naga. 2003;26(3):27.

[R18] Xu T, Zhang XH. Edwardsiella tarda: An intriguing problem in aquaculture. Aquaculture. 2014 Jul 20;431:129-35.

